# Duration of dual antiplatelet therapy in acute coronary syndrome

**DOI:** 10.1136/heartjnl-2016-309871

**Published:** 2017-03-01

**Authors:** Simon John Wilson, David E Newby, Dana Dawson, John Irving, Colin Berry

**Affiliations:** 1 British Heart Foundation Centre for Cardiovascular Science, New Royal Infirmary of Edinburgh, Edinburgh, UK; 2 Department of Cardiology, Ninewells Hospital, Dundee, UK; 3 British Heart Foundation Glasgow Cardiovascular Research Centre, University of Glasgow, Glasgow, UK; 6 Department of Cardiovascular Medicine, School of Medicine, Medical Sciences and Nutrition, University of Aberdeen, Aberdeen

**Keywords:** Acute coronary syndromes, Coronary artery disease, Diseases

## Abstract

Despite a large volume of evidence supporting the use of dual antiplatelet therapy in patients with acute coronary syndrome, there remains major uncertainty regarding the optimal duration of therapy. Clinical trials have varied markedly in the duration of therapy, both across and within trials. Recent systematic reviews and meta-analyses suggest that shorter durations of dual antiplatelet therapy are superior because the avoidance of atherothrombotic events is counterbalanced by the greater risks of excess major bleeding with apparent increases in all-cause mortality with longer durations. These findings did not show significant heterogeneity according to whether patients had stable or unstable coronary heart disease. Moreover, the potential hazards and benefits may differ when applied to the general broad population of patients encountered in everyday clinical practice who have markedly higher bleeding and atherothrombotic event rates. Clinicians lack definitive information regarding the duration of therapy in patients with acute coronary syndrome and risk scores do not appear to be sufficiently robust to address these concerns. We believe that there is a pressing need to undertake a broad inclusive safety trial of shorter durations of therapy in real world populations of patients with acute coronary syndrome. The clinical evidence would further inform future research into strategies for personalised medicine.

## Introduction

A ruptured or eroded coronary atherosclerotic plaque is the principal underlying cause of an acute coronary syndrome. The greatest ‘at risk’ period is during this early phase of plaque instability and healing, with recurrent event rates peaking in the first month. By 3 months, the plaque has usually stabilised, healed and subsequent event rates return to the background rates seen in patients with stable coronary heart disease.^1–3^ Indeed, beyond 3 months, recurrent events commonly occur on plaques at other sites within the coronary circulation.^3^ From first principles, the first 3 months is the most critical time for interventions to reduce recurrent cardiovascular events after an acute coronary syndrome (ACS). This is consistent with event rates seen in all clinical trials of patients with acute coronary syndrome: an initial time-varying high event rate that reverts to a consistent linear lower event rate from 3 months onwards (table 1).^1 2 4 5^

**Table 1:** Temporal relationship with the clinical benefits of clopidogrel therapy

**Time interval** **(months)**	Primary endpoint*		**ARR** **(%)**	**RRR** **[95% CIs] (%)**	**NNT** **(per month)**
	**Clopidogrel** **(%)**	**Placebo** **(%)**			
**CURE trial**					
0–1	4.3	5.5	1.2	22 [9, 32]	84
1–3	1.8	2.5	0.8	32 [13, 46]	240
3–6	1.8	1.8	0.0	4 [−27, 27]	5174
6–9	1.3	1.4	0.1	6 [–34, 34]	3171
9–12	1.1	1.3	0.2	14 [−32, 44]	1600
0–12	10.3	12.6	2.4	19	507
**CHARISMA trial**					
0–28	6.8	7.3	0.5	7*	5591
*Subgroup of patients with clinically evident atherosclerotic disease*					
0–28^‡^	6.9	7.9	1.0	12	2800

*Primary endpoint—cardiovascular death, myocardial infarction and stroke.

ARR, absolute risk reduction; CI, confidence intervals; CVD, cardiovascular death; MI, myocardial infarction; NNT, number needed to treat; RRR, relative risk reduction.

### Antiplatelet therapy

**Figure 1:**
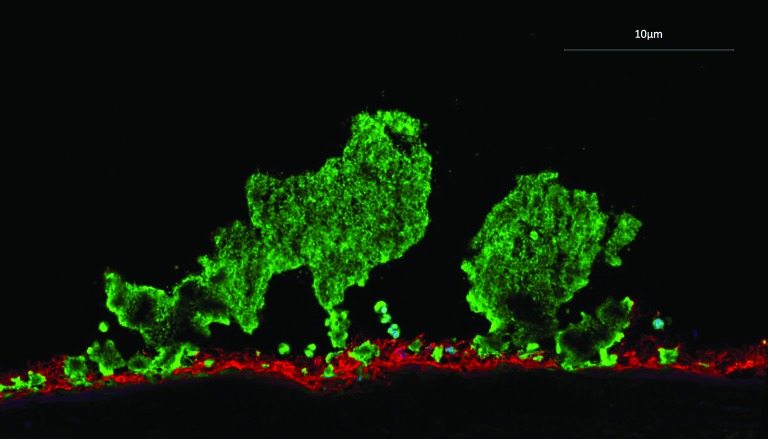
Immunofluorescent staining of human thrombus (platelets, green; fibrin, red) formed at high shear stress.

In an acute coronary syndrome, thrombus formation occurs under conditions of high shear stress and is principally driven by platelet aggregation (figure 1). This dominance of platelet aggregation during intracoronary thrombus formation reflects the dramatic effects that antiplatelet therapies have on clinical outcomes (table 2). Aspirin was the first antiplatelet therapy which induced a halving in event rates in patients with acute coronary syndrome:^6 7^ such a large effect size has rarely been surpassed in other domains of cardiology.

**Figure 2:**
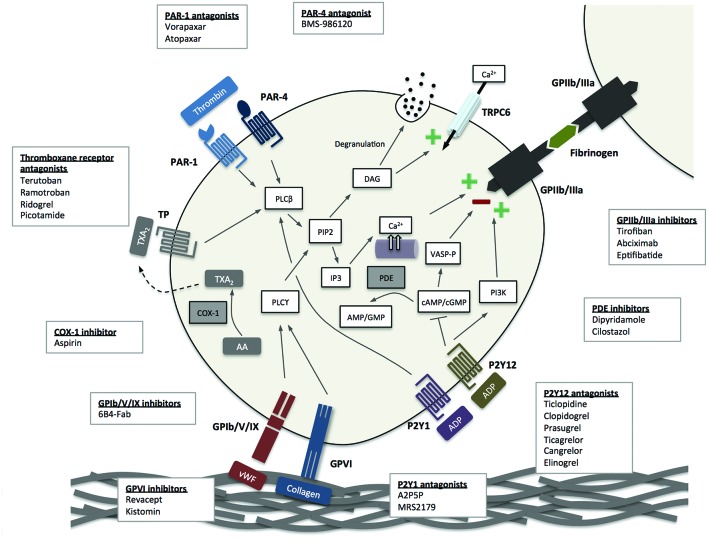
Platelet activation pathways and sites targeted by current and novel antiplatelet agents. Arachidonic acid; ADP, adenosine diphosphate; c, cyclic; Ca2+, calcium; AMP, adenosinemonophosphate; COX-1, cyclo-oxygenase-1; DAG, diacylglycerol; GMP, guanosine monophosphate; GP, glycoprotein; IP3: inositol trisphosphate; PAR, protease activated receptor; PDE, phosphodiesterase; PI3K,phosphatidylinositol 3-kinase; PIP2: phosphatidylinositol 4,5-bisphosphate; PLC, phospholipase C; TP, thromboxane receptor; TRPC, transient receptor potential channel; TXA2, thromboxane A2; vWF, von Willebrand factor.

Given aspirin’s remarkable success, it is perhaps unsurprising that adjunctive antiplatelet therapies have been investigated to build on these benefits, especially as there are multiple mechanisms of platelet activation beyond the cyclo-oxygenase pathway (figure 2). However, as platelets are essential to primary haemostasis, there is a balance between reducing the incidence of future cardiovascular events and causing harm from an increased risk of bleeding. The P2Y12 receptor antagonists are a class of drugs that have gained widespread acceptance since they appear to provide additional thrombotic protection at the expense of modest increases in bleeding. Their use is principally associated with reductions in recurrent myocardial infarction^1 4 5 8^ and in a few trials, reductions in cardiovascular events and mortality.^5 8^ Other antiplatelet therapies (figure 2) are available but have variable net clinical benefit and for the purposes of this review, we will consider only dual antiplatelet therapy (DAPT) with aspirin and P2Y12 receptor antagonism.

**Table 2:** Major trials of antiplatelet agents in acute coronary syndrome ± unstable angina

**Antiplatelet agent**	**Mechanism of action**	**Trial**	**Comparison**	**(Primary) end point**	**Risk reduction** **(time point)**
Aspirin	COX-1 inhibitor	The ISIS-2 collaborators (ISIS-2), 1979 The Risk Group, 1990^51^ Lewis *et al*, 1983^52^ Cairns *et al*, 1985^53^	aspirin versus placebo aspirin versus placebo aspirin versus placebo aspirin versus placebo	Vascular mortality MI or death MI or death MI or death	23% (5 weeks) 74% (3 months) 51% (3 months) 51% (2 years)
Ticlopidine	P2Y12 antagonist	Scrutinio *et al* (STAMI), 2001	aspirin versus ticlopidine	Death, MI, stroke or angina	ns (6 months)
Clopidogrel	P2Y12 antagonist	Bertrand *et al* (CLASSICS), 2000 Yusuf *et al* (CURE), 2001 The COMMIT Group (COMMIT), 2005 Sabatine *et al* (CLARITY), 2005	aspirin + clopidogrel versus aspirin + ticlopidine aspirin + clopidogrel versus aspirin aspirin + clopidogrel versus aspirin aspirin + clopidogrel versus aspirin	Cardiac death, MI, or TLR CV death, MI or stroke Death, MI or stroke CV death, MI or urgent TVR	ns (30 days) 20% (1 year) 9% (discharge or 28 days) 20% (30 days)
Prasugrel	P2Y12 antagonist	Wiviott *et al* (TRITON), 2007	aspirin + prasugrel versus aspirin + clopidogrel	CV death, MI or stroke	19% (1 year)
Ticagrelor	P2Y12 antagonist	Steg *et al* (PLATO), 2010	aspirin + ticagrelor versus aspirin + clopidogrel	CV death, MI or stroke	13% (1 year)
Dipyridamole	PDE inhibitor	The PARIS Research Group (PARIS-1), 1980 White *et al*, 1995^54^	aspirin + dipyridamole versus aspirin aspirin + dipyridamole versus aspirin	Cardiac death or MI Prevention of late reocclusion	ns (20 months) ns (1 year)
Cilostazol	PDE inhibitor	Lee *et al* (DECLARE-LONG II), 2011	Cilostazol + standard care versus standard care	In-stent late loss	18% (8 months)
Abciximab	GPIIb/IIIa inhibitor	The EPIC Investigators (EPIC), 1994 The CAPTURE Investigators (CAPTURE), 1997 Kastrati *et al* (ISAR-REACT 2), 2006 Simoons *et al* (GUSTO IV-ACS), 2001 Kastrati *et al* (IASR-REACT 4), 2011	12-hour infusion versus placebo 24-hour infusion versus placebo 12-hour infusion versus placebo 24-hour or 48-hour infusion versus placebo Abciximab + heparin versus bivalirudin	Death, MI or urgent revascularisation Death, MI or urgent revascularisation Death, MI or urgent TVR Death or MI Death, MI, urgent TVR or major bleeding	35% (30 days) 29% (30 days) 24% (30 days) ns (30 days) ns (30 days)
Eptifibatide	GPIIb/IIIa inhibitor	The PURSUIT Investigators (PURSUIT), 1998	Bolus and 72-hour infusion versus placebo	Death or MI	10% (30 days)
Tirofiban	GPIIb/IIIa inhibitor	The PRISM investigators (PRISM), 1998 The PRISM investigators (PRISM-PLUS), 1998	Bolus and 48-hour infusion versus placebo Bolus and 72-hour infusion versus placebo	Death, MI, refractory ischaemia or readmission for UA Death, MI or refractory ischaemia	ns (30 days) 17% (30 days)
Vorapaxar	PAR-1 antagonist	Tricoci *et al* (TRACER), 2012	vorapaxar + standard care versus standard care	CV, death, MI, readmission with ischaemia, urgent revascularisation	ns (median 502 days)

COX-1, cyclo-oxygenase-1; CV, cardiovascular; GP, glycoprotein; MI, myocardial infarction; PAR-1, protease-activated receptor-1; PDE, phosphodiesterase; TLR, target lesion revascularisation; TVR, target vessel revascularisation; UA, unstable angina.

### Dual antiplatelet therapy

The benefit of dual antiplatelet therapy following an acute coronary syndrome was established by the CURE,^1^ COMMIT/CCS-2^8^ and CLARITY-TIMI 28^9^ trials. Combined aspirin and clopidogrel therapy reduced the 1-year incidence of cardiovascular events by approximately 20% compared with aspirin alone. More potent and consistent P2Y12 receptor inhibition with either prasugrel or ticagrelor was superior to clopidogrel in the subsequent TRITON^4^ and PLATO^5^ trials.

The evidence for dual antiplatelet therapy in patients with stable coronary heart disease is less distinct. In the CHARISMA trial,^2^ the addition of clopidogrel to aspirin in patients with established cardiovascular disease or at high risk of clinical atherosclerotic disease did not reduce cardiovascular events and was associated with an increase in severe bleeding. There was, however, a suggestion of improved outcomes in patients with established atherothrombotic disease, particularly those with a history of myocardial infarction. The PEGASUS-TIMI 54^10^ trial compared aspirin monotherapy to a combination of aspirin and ticagrelor in patients with a previous myocardial infarction and at least one additional high-risk factor. At a mean of 33 months, ticagrelor (60 mg) reduced the incidence of cardiovascular death, myocardial infarction or stroke (7.77% vs 9.04%) at the expense of increased thrombolysis in myocardial infarction (TIMI) major bleeding (2.30% vs 1.06%) and a neutral effect on overall mortality. On the basis of these trials, combination antiplatelet therapy would appear to confer only a small ischaemic benefit at the cost of a significant bleeding risk. European^11^ and North American^12^ guidelines therefore do not recommend dual antiplatelet therapy in patients with stable atherothrombotic disease but acknowledge that with careful consideration, combined antiplatelet therapy may be beneficial in some high-risk patients.

### Duration of dual antiplatelet therapy: clinical trials

Current European^13^ and North American^12^ guidelines advise continuing dual antiplatelet therapy for 1 year following an acute coronary syndrome. These recommendations are made on the basis of early studies^4 5 14 15^ demonstrating a sustained increased risk of thrombotic complications, including stent thrombosis and spontaneous cardiovascular events, beyond 6 months. However, the greatest absolute reductions in cardiovascular events with dual antiplatelet therapy are seen in the first 3 months (table 1) and since these studies, advances in drug-eluting stent technology have led to a substantially reduced incidence of late (>30 days) and very late (>1 year) stent thrombosis.^16^

**Figure 3:**
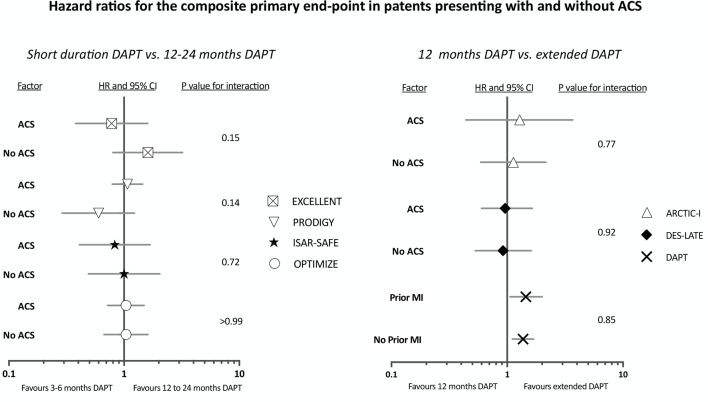
Hazard ratios for the composite primary end-point from sub-group analyses of patents presenting with and without an acute coronary syndrome. EXCELLENT trial (n=1443), 6 vs 12 months, patients presenting with ACS = 52% of the study population; PRODIGY trial (n=2013), 6 vs 24 months, patients presenting with ACS subgroup = 74% of study population; ISAR-SAFE trial (n=4000), 6 vs 12 months, patients presenting with ACS = 40% of the study population; OPTIMIZE trial (n=3119), 3 vs 12 months, patients presenting with ACS = 37% of the study population; ARTIC INTERRUPTION (n=1259), 12 vs 30 months, patients presenting with ACS = 26% of the study population; DES-LATE (n=5045), 12 vs 24 months, patients presenting with ACS = 61% of the study population and DAPT (n=9961), 12 vs 30 months, patients presenting with ACS = 43% of the study population.

In recent trials of patients treated with newer generation drug-eluting stents, shorter durations of dual antiplatelet therapy (3 months to 6 months) were non-inferior to 12^17–22^ months or 24^23^ months of treatment with regard to etiher a composite of cardiovascular events or cardiovascular events plus major bleeding. Moreover, all of these trials included patients with an acute coronary syndrome (range 23% to 74% of study population) and in those who undertook prespecified subgroup analyses, no heterogeneity in treatment effect between stable and unstable coronary artery disease was observed (figure 3).

Beyond 12 months, there remains a residual risk of local and systemic atherothrombotic complications^24^ and a number of studies have examined whether extended dual antiplatelet therapy (>12 months) following percutaneous coronary intervention may be beneficial. In the DES-LATE^25^ and ARCTIC-INTERRUPTION trials,^26^ prolonged treatment with dual antiplatelet therapy (18–30 months vs 12 months) neither reduced the incidence of cardiovascular events nor increased the risk of major bleeding. Among those patients presenting with an acute coronary syndrome, primary and secondary ischaemic end points did not differ from the global treatment population. In the DAPT trial,^27^ the largest and only double-blinded study, extended dual antiplatelet therapy (30 months vs 12 months) reduced the risk of major adverse cardiovascular and cerebrovascular events (4.3% vs 5.9%), myocardial infarction (2.1% vs 4.1%) and stent thrombosis (0.4% vs 1.4%) but at a cost of increased moderate or severe bleeding (2.5% vs 1.6%) and a borderline rise in all-cause mortality (2.0% vs 1.0%; p=0.05). Treatment effect did not differ between patients with or without a history of myocardial infarction for any of the co-primary end points including bleeding (figure 3).

### Duration of dual antiplatelet therapy: systematic reviews and meta-analyses

Meta-analyses of trials using dual antiplatelet therapy in patients receiving intracoronary stents have compared short (3–6 months), 12-month and prolonged (>12 months) durations of therapy.^28 29^ Longer treatment periods reduced the incidence of myocardial infarction and stent thrombosis but at a cost of increased major bleeding and with a tendency to increase overall mortality because of an increase in non-cardiovascular death. However, the majority of patients included in these analyses had stable coronary artery disease and few patients with acute coronary syndrome were treated with ≤6 months of dual antiplatelet therapy. In a recent meta-analysis^30^ that included only patients with a history of acute coronary syndrome, prolonged dual antiplatelet therapy reduced the risk of cardiovascular death (RR 0.85; 95% CI: 0.74 to 0.98, p=0.03) without an increase in non-cardiovascular death (RR 1.03, 95% CI 0.86 to 1.23; p=0.76) or all-cause mortality (RR 0.92, 95% CI 0.83 to 1.03).

### Atherothrombotic risk

The optimal duration of dual antiplatelet therapy is dependent on the balance between preventing future atherothrombotic events and the increased risk of bleeding from continued treatment. Following an acute coronary syndrome, predictors of atherothrombotic risk include ST deviation, diabetes mellitus, smoking status, left ventricular ejection fraction, stent type, number of stents and complexity of coronary artery disease. Subgroup analyses have attempted to identify if any of these factors influence outcomes with regard to duration of dual antiplatelet treatment.

In the EXCELLENT trial,^22^ there was a threefold increase in the incidence of cardiovascular events (p<0.001 for interaction) among pat￼ients with diabetes mellitus treated with 6 months as compared with 12 months of dual antiplatelet therapy. In the ISAR-SAFE trial,^20^ rates of death, myocardial infarction, stent thrombosis, stroke or TIMI major bleeding tended to be higher in patients aged <67 years and lower in patients aged ≥67 years with 6 months compared with 12 months of dual antiplatelet treatment (p=0.03). These differences were driven by ischaemic complications rather than bleeding events. In the DAPT trial, men were nearly five times less likely to suffer from stent thrombosis if dual antiplatelet therapy was extended beyond 12 months (p=0.04) and the reduction in major adverse cardiovascular and cerebrovascular events with prolonged therapy (30 months) was greater in those treated with prasugrel (vs clopidogrel; p=0.03) or a first-generation drug-eluting stent (p=0.048). Similar trends were observed in DES-LATE^25^ and ARCTIC-INTERRUPTION.^26^

Audit of the British Cardiovascular Society Intervention database indicates that in 2013/2014, a half of all percutaneous coronary intervention procedures were associated with residual disease (≥1 stenosis of >50% severity), fulfilling the criteria for incomplete revascularisation. Patients with acute coronary syndrome and incomplete revascularisation have a residual burden of coronary disease that is a substrate for recurrent plaque rupture, coronary thrombosis and future cardiac events.^31 32^ Prolonged dual antiplatelet treatment may mitigate this risk but whether this translates to a more favourable risk-to-benefit balance for patients with incomplete revascularisation remains an area for future research.

### Bleeding and total mortality

Large registries and trials have shown that major bleeding is associated with an increase in mortality that could potentially negate the benefits of dual antiplatelet therapy in acute coronary syndrome.^33–36^ Importantly, these bleeding risks are not confined to the initial hospitalisation phase.^33 35^ The association between bleeding and mortality has been a consistent feature of acute coronary syndrome trials irrespective of whether the intervention being assessed and improvements in outcome are seen with interventions that are associated with a lower bleeding risk. For example, in the OASIS-5 trial,^33^ fondaparinux had similar antithrombotic benefits to enoxaparin but was associated with lower rates of major bleeding and marked reductions in all-cause mortality. Similar benefits have also been reported for randomised controlled trials of arterial access sites in patients treated with an invasive strategy for either ST-segment^37^ or non-ST-segment^38^ myocardial infarction. Again, because radial artery access was associated with less bleeding, overall all-cause mortality was lower.^37 38^ There have been various mechanisms proposed for the link between bleeding and mortality that include rebound hypercoagulability, discontinuation of antithrombotic treatments, inflammation and ischaemia.^39^ The European Society of Cardiology Working Group on Thrombosis has called for clinical trials to address bleeding in acute coronary syndrome including the exploration of the duration of dual antiplatelet therapy.^39^

### Duration uncertainty

Currently there are variations in local and regional dual antiplatelet therapy practices that are confusing for patients, primary care physicians and cardiologists. Indeed, while European^13^ and North American^12^ guidelines recommend dual antiplatelet therapy for 12 months after an acute coronary syndrome, both acknowledge that shorter or longer durations may be appropriate. Duration of therapy is seen as a major priority for future research by numerous national and international guideline committees as well as having considerable financial implications, especially for the latest generation of P2Y12 receptor antagonists. However, major pharmaceutical companies have to date not funded trials comparing shorter (<12 months) durations of dual antiplatelet therapy, since, arguably, it may not be in their commercial interest to do so.

For clinicians and healthcare providers, there remains much uncertainty regarding the default duration of dual antiplatelet therapy for most patients with acute coronary syndrome. Current guidelines are largely based on evidence that predates potentially important technological advances, including second-generation drug-eluting stents, while in recent trials, only a minority of patients presented with an acute coronary syndrome and many of these studies were underpowered to detect differences due to low event rates. Selected populations included in randomised controlled trials have lower rates of bleeding and non-cardiovascular death than the general population (table 3), since patients with any history of bleeding or major comorbidity were specifically excluded from such trials. On the other hand, shorter durations of dual antiplatelet therapy may expose medically managed patients (such as due to complex disease) to an increased risk of atherothrombotic events. This is because stenting in acute coronary syndrome confers protection against atherothrombotic events^40 41^ and in the vast majority of clinical trials, only patients who underwent stent implantation were included. There is therefore a major concern that the evidence to date has been extrapolated to a broader population with a higher risk of both atherothrombotic events and adverse outcomes from bleeding. Accordingly, this has left uncertainty as to the relative benefits and risks of one period of treatment versus another in real-world patients with acute coronary syndrome.

**Table 3:** Temporal relationship with the clinical benefits of clopidogrel therapy

**Outcome**	**CURE** (clopidogrel)	**TRITON** (prasugrel)	**TRILOGY** (prasugrel)	**PLATO** (ticagrelor)	**Randomised controlled trial average**	**Scotland** **2006–2010** ^*^ (clopidogrel)
**All-cause mortality**	5.8%	3.0%	8.3%	4.5%	5.40%	25.4%
**Cardiovascular death**	5.1%	2.1%	6.6%	4.0%	4.45%	17.8%
**Non-cardiovascular death**	0.7%	0.9%	1.7%	0.5%	0.95%	7.6%
**Fatal bleeding**	0.2%	0.4%	0.2%	0.3%	0.28%	0.9%

*From Information and Statistics Division of NHS Scotland.

Increased thrombin generation^42^ and platelet reactivity^43^ have been demonstrated for up to 2 years following a plaque rupture myocardial infarction. This may explain potentially more favourable outcomes with longer durations of dual antiplatelet therapy in patients with acute coronary syndrome as compared to those with stable ischaemic heart disease undergoing percutaneous coronary intervention. However, switching from dual antiplatelet treatment to monotherapy (usually aspirin alone) is associated with a rebound prothrombotic effect, especially with regard to an excess of stent thrombosis.^10 27^ In the DAPT trial,^27^ this phenomenon occurred irrespective of the timing of switching to monotherapy. Thus, unless dual antiplatelet therapy is continued indefinitely, there will remain a small persistent short-term 3-month risk of rebound stent thrombosis and myocardial infarction following the transition from dual to single antiplatelet therapy.

Newer generation P2Y12 inhibitors provide more effective antithrombotic protection than clopidogrel but at the cost of increased bleeding. Given the greater expense of these agents and the marked temporal decline in thrombotic risk that is evident over the first few months, an early ‘switch’ from ticagrelor or prasugrel to clopidogrel after 1 month to 6 months has been advocated. While evidence from small cohort^44^ and pharmacodynamic studies^45^ suggest such an approach may be safe and reduce bleeding events, an overall lack of data limits any meaningful recommendations. TROPICAL-ACS (NCT 01959451) is an ongoing clinical trial investigating whether a switch to clopidogrel treatment after 1 week of prasugrel is non-inferior to 12 months of standard treatment with prasugrel. Results from this and other similar randomised studies (SWAP-4, NCT 02287909) should provide insights into defining the best strategy for switching between P2Y12 antagonists.

### Personalised medicine

Generic recommendations for length of dual antiplatelet therapy derived from a protocolised intervention in clinical trials will inevitably expose some patients to an excessive duration of treatment and disadvantage other patients by withdrawing therapy that protects them from myocardial infarction. The critical clinical question is therefore: can individuals who are more or less likely to benefit from shorter or longer durations of treatment be identified? While several risk tools have been developed to help determine the future incidence of coronary thrombotic events and major bleeding episodes for an individual, the DAPT Score^46^ and PARIS registry risk Score^47^ were specifically designed to predict medium-term to long-term risks that are directly modified by continuing or interrupting dual antiplatelet therapy.

**Figure 4:**
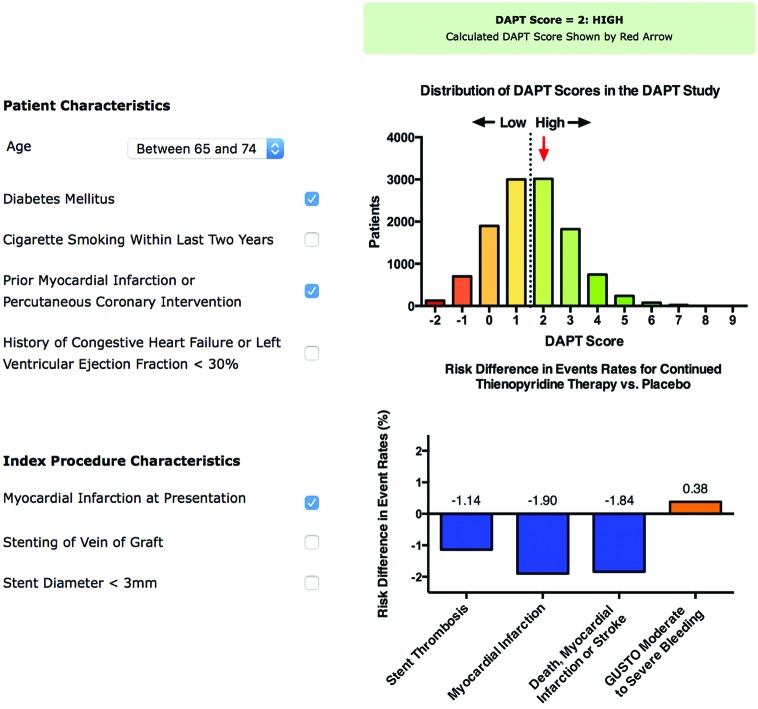
DAPT Score calculator for predicting risk/benefit of extending dual antiplatelet therapy from 12 months to 30 months.Available at www.daptstudy.org.

Using data from the DAPT trial, the DAPT Score was developed to determine the net clinical benefit of extending dual antiplatelet therapy from 12 months to 30 months (figure 4). Thirty-seven candidate variables were considered with 8 included in the final model. In the validation cohort the c-statistic was 0.64 for ischaemia and 0.64 for bleeding. The PARIS registry risk score was similarly developed but was based on prospective observational data. Model discrimination for ischaemia (c-statistic 0.65) and bleeding (c-statistic 0.64) events were comparable to the DAPT score. Thus while these and other similar risk / benefit tools may provide a step forward, they do not as yet offer sufficiently robust predictive value for everyday clinical use.

Variables included in risk tools tend to predict both bleeding and myocardial infarction. High platelet reactivity was originally reported to identify patients at an increased risk of future atherothrombotic events.^48^ However, subsequent studies have failed to show an association between platelet reactivity and atherothrombotic outcomes^49^ or demonstrate that dose adjustment on the basis of on-treatment platelet reactivity is beneficial.^50^ Routine platelet testing is therefore not recommended in patients prescribed P2Y12 inhibitors and is unlikely to deliver a clinically useful prediction score. Further research is needed to define prediction tools with high discriminatory value in real-world patients, and the potential for net-clinical benefit according to the length of dual anti-platelet therapy.

## Conclusions

It has been 15 years since the CURE trial demonstrated the benefit of dual antiplatelet therapy following an acute coronary syndrome and yet the optimal duration remains uncertain. With regard to thrombotic complications, recent clinical trials and meta-analyses suggest that with newer generation drug-eluting stents, 3 months to 6 months of dual antiplatelet therapy is non-inferior to 12 months of treatment. Prolonged treatment (>12 months) reduces the risk of stent thrombosis, myocardial infarction and possibly cardiovascular death but at the cost of increased major bleeding and with no net mortality benefit. However, these potential hazards and benefits of intervention may differ when applied to the general broad population of patients encountered in everyday clinical practice who have higher bleeding and atherothrombotic event rates.

While ongoing randomised clinical trials may address some of the residual uncertainties in select subgroups, we believe there is a pressing need to undertake a broad inclusive trial of shorter durations of therapy in broad populations of patients with acute coronary syndrome. Such a trial will need to be able to explore specific subgroups, such as those who are medically managed, undergo percutaneous coronary intervention or have coronary artery bypass graft surgery, as well as enable better identification of atherothrombotic and bleeding risks from real world data to inform a more personalised approach to decisions regarding treatment duration.
